# Explaining the effects of a multifaceted intervention to improve inpatient care in rural Kenyan hospitals -- interpretation based on retrospective examination of data from participant observation, quantitative and qualitative studies

**DOI:** 10.1186/1748-5908-6-124

**Published:** 2011-12-02

**Authors:** Mike English, Jacinta Nzinga, Patrick Mbindyo, Philip Ayieko, Grace Irimu, Lairumbi Mbaabu

**Affiliations:** 1 KEMRI-Wellcome Trust Research Programme, P.O. Box 43640, Nairobi 00100, Kenya; 2 Department of Paediatrics, University of Oxford, John Radcliffe Hospital, Oxford, UK; 3 Department of Paediatrics and Child Health, College of Health Sciences, University of Nairobi, Nairobi, Kenya

## Abstract

**Background:**

We have reported the results of a cluster randomized trial of rural Kenyan hospitals evaluating the effects of an intervention to introduce care based on best-practice guidelines. In parallel work we described the context of the study, explored the process and perceptions of the intervention, and undertook a discrete study on health worker motivation because this was felt likely to be an important contributor to poor performance in Kenyan public sector hospitals. Here, we use data from these multiple studies and insights gained from being participants in and observers of the intervention process to provide our explanation of how intervention effects were achieved as part of an effort to better understand implementation in low-income hospital settings.

**Methods:**

Initial hypotheses were generated to explain the variation in intervention effects across place, time, and effect measure (indicator) based on our understanding of theory and informed by our implementation experience and participant observations. All data sources available for hospitals considered as cases for study were then examined to determine if hypotheses were supported, rejected, or required modification. Data included transcriptions of interviews and group discussions, field notes and that from the detailed longitudinal quantitative investigation. Potentially useful explanatory themes were identified, discussed by the implementing and research team, revised, and merged as part of an iterative process aimed at building more generic explanatory theory. At the end of this process, findings were mapped against a recently reported comprehensive framework for implementation research.

**Results:**

A normative re-educative intervention approach evolved that sought to reset norms and values concerning good practice and promote 'grass-roots' participation to improve delivery of correct care. Maximal effects were achieved when this strategy and external support supervision helped create a soft-contract with senior managers clarifying roles and expectations around desired performance. This, combined with the support of facilitators acting as an expert resource and 'shop-floor' change agent, led to improvements in leadership, accountability, and resource allocation that enhanced workers' commitment and capacity and improved clinical microsystems. Provision of correct care was then particularly likely if tasks were simple and a good fit to existing professional routines. Our findings were in broad agreement with those defined as part of recent work articulating a comprehensive framework for implementation research.

**Conclusions:**

Using data from multiple studies can provide valuable insight into how an intervention is working and what factors may explain variability in effects. Findings clearly suggest that major intervention strategies aimed at improving child and newborn survival in low-income settings should go well beyond the fixed inputs (training, guidelines, and job aides) that are typical of many major programmes. Strategies required to deliver good care in low-income settings should recognize that this will need to be co-produced through engagement often over prolonged periods and as part of a directive but adaptive, participatory, information-rich, and reflective process.

## Background

Dramatic improvements in child, newborn and maternal survival by 2015 are major global (millennium) development goals (MDGs 4 and 5 [1]). However, one of the most pressing challenges of the current era remains the question 'how can we best achieve implementation of high-quality essential interventions at scale within health systems?' [2,3]. Achieving this goal demands an understanding of implementation that goes beyond the 'mean effect size' emerging from controlled experiments.

Much of the research available examining delivery of interventions in low-income settings is from outpatient or primary care settings [4,5]. Some major evaluations have spanned several countries and measured and explored the likely causal pathway to effects (a good example being the Integrated Management of Childhood Illness Multi-Country Evaluation work [6-9]). However, rural hospital care has been the subject of less research. Although their value has been recognized since the Alma Ata declaration [10], reports available indicate that the services they provide are often poor, in areas including surgical, maternal, paediatric, and neonatal care [11-16]. We also have only limited understanding of them as health system contexts within which essential interventions must be implemented. Yet, what we observe at patient level is clearly shaped by actors and policies at multiple levels within a health system [17,18].

However examining the range of factors influencing care is likely to require different methodological approaches. For such reasons it has been recommended that qualitative research should be conducted alongside trials or evaluations of complex interventions to facilitate understanding of effects in different contexts [19,20]. The potential benefits of mixing research methods encouraged us to develop a platform of work around a founder study testing an intervention to improve essential paediatric hospital services in Kenya. Here, we attempt to synthesize the findings of the quantitative and qualitative research we undertook over a period of four years. Our primary aim is to offer explanations for why the intervention did or did not produce desired effects.

## Methods

We planned a cluster randomized trial of a multifaceted intervention aimed at improving paediatric inpatient care. The rationale for a multifaceted approach is provided elsewhere [21], but largely reflected the knowledge that single interventions, for example training, often have little or no effect on observed practice [22]. Thus, as well as the concrete inputs within the intervention packages (training, guidelines, job aides), the strategy comprised a low-intensity, normative-reeducative approach to organizational change [23], including: re-setting norms and values (linked to creating a new 'vision' for and commitment to better patient care) and a focus on partnership, local problem solving, and empowerment. This founder study is described in brief in Additional File 1 with full descriptions of the design and intervention effects provided elsewhere [21,24,25]. This trial also provided the framework for four, largely qualitative or contextual pieces of work (see Additional File 2 for summary) also described in full elsewhere [26-29]. Here, the data generated by all these studies form the primary resource material for the synthesis and interpretation now presented. All these data were collected during studies receiving national scientific and ethical approval with individual informed consent where appropriate.

Classifying the strategy in line with an emerging typology for mixed methods research [30] the dominant disciplinary approach was thus quantitative, with qualitative research aimed primarily at strengthening explanatory insights and allowing likely generalisability to be considered. However, our qualitative enquiry was not aimed at providing data simply to complement interpretation of quantitative effect measures (for a recent example of this see [31]), suggested as one form of mixed methods research [32]. Rather we undertook qualitative studies that we felt could stand alone as discrete pieces of work, attempting to keep some distance between specific streams of work while maintaining a disciplined approach to qualitative analysis to promote objectivity and legitimacy.

The available work is perhaps therefore reasonably described as multi-method with multiple, parallel but linked strands of enquiry tackling an overarching question [33]. Yet even this description is somewhat misleading. A metaphor of strands implies distinct boundaries between pieces of work even if subsequently combined in an integrated analysis. The team approach we adopted instead produced multiple streams of research, with scope for some mixing of ideas, even though distinct currents were at work. In addition, some members of the team were involved in delivery of the intervention throughout the project and brought to the team prior insights from a history of research on delivering services in Kenyan hospitals [11,34]. Thus, significant elements of participatory action research informed intervention delivery, and the analyses and interpretations we present are infused by long-term participant observation gained from experience as external supervisors to the study hospitals [35,36]. Here, therefore, we acknowledge and employ our position as engaged with and exposed to the multiple currents of enquiry and as co-producers of the intervention itself to attempt, deliberately, to draw together the streams of work and offer our interpretation of how the intervention caused effects. In doing this, our focus has been on tackling the following major question: 'Why did performance assessed as uptake of, or adherence to recommended best practices vary, often dramatically, between practices (for which performance indicators were developed), between hospitals and between the full and partial intervention groups?'

### Approach to analysis

The analytic strategy adopted could be described as the 'ethnography of an intervention' and methodologically is similar to the idea of 'following a thread' presented by O'Cathain [37]. Thus, a primary idea or theory was identified, in this case to explain the quantitative findings, and followed within and across the other components of our research. This was undertaken by one investigator (ME) who developed an initial set of theories after scrutinising performance patterns for the 14 key quantitative indicators [24]. This process was informed by insights gained from being an originator of the project, a survey team leader, supervisor, and focal point for feedback, as well as an investigator closely involved in the previously reported qualitative analyses. Theory was thus developed from a specific standpoint, that of an informed member of the implementing team able to observe interactions between implementers, and study hospitals and their staff over a period of more than three years. Initial low-level theories were listed and then examined by returning to the primary data and examining them for congruence with the hypotheses and by looking for divergent or extreme cases. Hypotheses were then revised, abandoned, or merged into larger thematic explanations in an iterative process until those that appeared both best supported by the data and most useful in providing explanations remained. The approach therefore is perhaps most similar to a cross-case analysis [38] in which we aimed to derive a conceptual understanding of variation in intervention effects that went beyond the particularities of a specific hospital. As emerging cross-case explanatory theory was developed, this was further refined through reflection and discussion that engaged the wider team of investigators to examine its appropriateness and reduce redundancy.

Throughout this work initial theory building and the theoretical positions of the team were informed by the simple, original 'layered' conceptual framework that formed the basis for the intervention study [21] and multiple theories on factors influencing healthcare provision including: diffusion of innovations [39]; barriers to uptake of guidelines [40]; the theory of planned behavior [41]; motivation and worker performance [4,42,43]; clinical microsystems [44]; organizational culture; and transforming systems [17,45]. We recognize that our framework is bounded by this body of theory and knowledge available to the research team. Further, our framework is limited as data from the highest system or policy level were not collected. Finally we have not tested our ideas or this framework by returning to the original hospital sites to discuss them with staff, nor have we evaluated its ability to explain other interventions. We thus offer a form of mid-level theory [46] relevant to the hospitals and form of intervention studied, but one that would need additional scrutiny before any wider value is confirmed, although we have 'mapped' our intervention and those factors related to its achievements against the consolidated framework for advancing implementation science [47] (see Additional File 3).

### Role of the funding source

Funds from a Wellcome Trust Senior Fellowship awarded to Dr. Mike English (#076827) supported the founder study and linked qualitative work, while funds from a Wellcome Trust Strategic Award for training (#084538) provided support to PM and LM. The funders had no role in the design, conduct, analyses or writing of this report or in the decision to submit for publication.

## Results

We have presented elsewhere a full report on the quantitative findings from our research [24]. Here, we present for fourteen key indicators the mean improvement in performance from baseline to eighteen months for full (intervention) or partial (control) intervention package groups, and the difference in improvement between these groups. In addition, we present the best and worst performance noted across the set of eight hospitals considered as a cross-section at the eighteen months endpoint (Table 1). These data demonstrate the variations in absolute levels of performance and magnitude of improvement that are the focus of interest. Not presented here are additional results from secondary analyses of the hospital study data. Of interest were findings suggesting that full intervention hospitals showed greater commitment to, and success in, solving local resource and organizational challenges [24]. However additional data also indicate relatively poor performance in most hospitals for practices related to newborn care and the management of severe malnutrition (unpublished data). Although best practices in these areas were also articulated in the guidelines that were disseminated, these conditions were not the primary focus of performance feedback to hospitals and are less commonly encountered by hospital clinicians in their day-to-day roles.

**Table 1:** Effectiveness of full intervention compared with partial intervention (control) 18 months after initiation for 14 primary outcome measures with full range of observed performance across the 8 hospitals at 18 months indicated by minimum and maximum performance values.

Indicator of quality of care (target 100%, maximum score or 0% where indicated by *)	End of Intervention		Mean change from baseline (%)		Difference-in-difference (%)	*95%CI* *(%)*	
	*Min*	*Max*	Intervention	Control			
Child's weight documented	***45%***	***97%***	25.2	42.2	-17.1	*-76.5*	*42.4*
Child's temperature documented	***1%***	***90%***	60.0	21.5	38.5	*3.42*	*73.5*
Average assessment score (range 0 - 1)	***0.38***	***0.97***	0.62	0.33	0.29	*0.04*	*0.55*
Proportion of pneumonia episodes with a severity classification	***16%***	***97%***	85.8	42.3	43.5	*-9.47*	*96.5*
Proportion of gentamicin prescriptions with once daily dose	***68%***	***97%***	87.4	70.9	16.5	*0.69*	*32.3*
Proportion of gentamicin prescriptions with daily dose < 4 mg/kg*	***0%***	***17%***	-22.8	-14.4	-8.36	*-40.0*	*23.2*
Proportion of gentamicin prescriptions with daily dose > = 10 mg/kg*	***1%***	***16%***	2.5	2.7	-0.20	*-9.44*	*9.04*
Proportion with adequate oxygen Prescriptions	***0%***	***78%***	37.0	-0.5	32.6	*-3.57*	*68.8*
Proportion of malaria episodes with a severity classification	***10%***	***97%***	82.4	38.6	43.8	*4.14*	*83.5*
Proportion of severe malaria with quinine loading	***31%***	***98%***	87.7	51.9	35.8	*-30.70*	*100*
Proportion of severe malaria with twice daily quinine maintenance dose	***34%***	***97%***	87.5	35.7	51.8	*27.6*	*75.9*
Proportion of severe malaria with quinine daily dose > = 40 mg/kg*	***0%***	***16%***	-6.3	-6.7	0.36	*-8.63*	*9.35*
Proportion of dehydration episodes with a severity classification	***68%***	***99%***	45.9	24.3	21.6	*-8.97*	*52.1*
Correct intravenous fluid prescription	***28%***	***83%***	59.9	25.6	34.3	*13.8*	*54.8*
***Outcome indicators***							
Proportion with Vitamin A Administered on Admission	***0%***	***75%***	31.4	-3.3	34.8	*-10.6*	*80.2*
Age appropriate documentation of immunisation status	***1%***	***28%***	47.3	4.5	42.8	*8.69*	*77.0*
Provider Initiated HIV testing among unknown HIV	***1%***	***75%***	20.8	2.8	17.9	*10.2*	*25.7*
Mean proportion of discharge counseling tasks performed (score range 0 - 4)	***0.64***	***3.55***	1.36	0.82	0.55	*-2.04*	*3.13*

**Figure 1:**
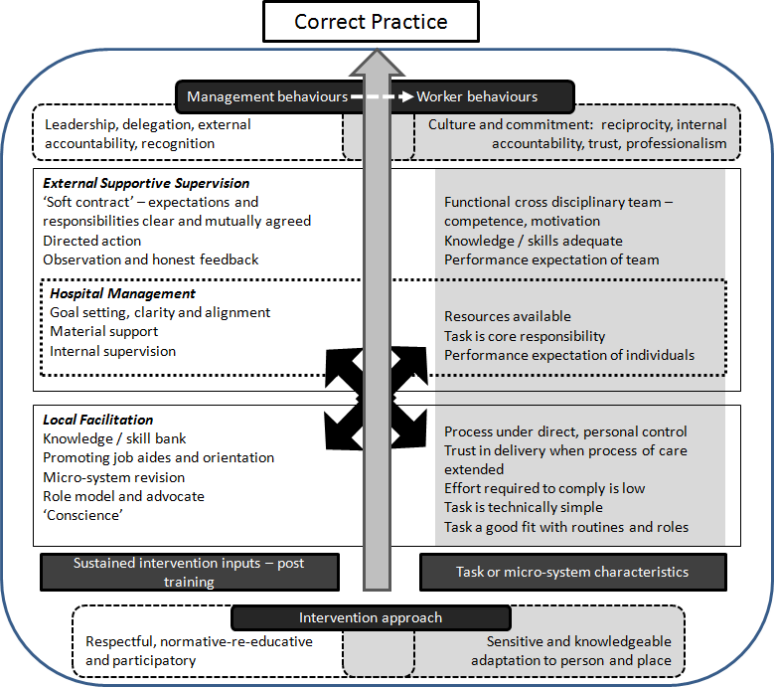
**Over-arching framework indicating how the full intervention package both produced overall effects and variability in effects**. The intervention approach included sustained inputs of supervision, feedback, and local facilitation. Where successful, it appeared able to engage hospital management as actors and influence the focus of action (External Support Supervision and Hospital Management tables) and way of acting of managers (Management Behaviours). These behaviours were greatly supported by facilitators acting as change agents (Local Facilitation). Success in organisational or procedural changes (left side of Figure) impacted to generate more positive worker behaviour (right side of Figure) and provided more supportive micro-systems within which care was provided. Correct practice by frontline workers was thus promoted particularly for simple, low-effort tasks recognised as key responsibilities within familiar roles.

Consistent with our analytic approach, we find that the normative-reeducative approach and aspects of the intervention that were sustained throughout the eighteen-month period, considered as operating at different organizational levels, are important in determining performance measured as correct care. The effectiveness of intervention inputs to promote positive changes was, however, modified by behaviours of people at different organizational levels, characteristics of the actual tasks required of health workers, and their micro-system. These relationships are summarized in Figure 1, noting that the nature of interactions between the implementing team, the actual hospital management, facilitators (in intervention sites), and front-line workers were co-produced by all parties and evolved over time. Similarly the hospital management, influenced to a degree by the implementing team and perhaps particularly the facilitator, had the chance to influence the micro-system within which front-line workers operated to establish what was expected and possible. Successful adoption of best practices therefore appeared where the implementing team, hospital management, and facilitator together provided leadership and supported a shift in organizational culture and commitment that helped motivate health workers and change their individual behaviours. Performance data provided by a credible, supportive source helped promote accountability and direct change efforts. Having presented such an overarching framework, we now consider some of the specific factors contributing to varied performance recognizing their multiple inter-relationships (Figure 1).

### External supportive supervision and local management

All hospitals were informed of the nature of the study and agreed to join before being assigned to full or partial intervention groups. However, meetings were held with the senior management teams of full intervention group hospitals around the time of introductory training and after the results of each six-month survey. Such meetings, less formal meetings with managers during supervisory visits, and the lines of communication maintained through hospital facilitators helped establish what was expected of hospitals in terms of performance improvement, and clarified management's responsibilities and expected actions. While these did not constitute formally constructed, written agreements, this dialogue helped develop a 'soft-contract' with partners aware of and, to a degree, committed to supporting improved care. Further effects of supportive supervision were dependent on additional mediating factors: the actual hospital managers and their internal leadership and relationships; managers' competing priorities; the personal relationships with supervisors; the credibility and acceptance of the performance feedback; and the balance achieved between praise and positive reinforcement in areas going well as well as de-motivation or defensiveness where progress was less obvious.

'They [senior managers] never even come to see how we work here, to ask what challenges we encounter, they don't even come..... So they never come to see how we are doing, they just depend on hear say and rumors, and may be they say we are doing good work because they have never heard complaints that we are not doing the work.'

'[The supervision] has assisted us in improvement, at all levels because there is no feedback that you have given us that we have not acted on, we have acted on all of them, but the resource factor, that is a problem. We may note something, but if we do not have the resources then that's it. The feedbacks have been very important to us, not just in helping us improve, but they have made us do a quick survey and like I told you we did a quick survey and we realized we need to talk to people about their attitudes.'

'If we are all stuck then we consult our pediatrician, because he really assists us. In fact you don't have to go to the office to ask them to call him for you, just call him on your phone and he will come immediately if he is within, and if he is not within he will look for somebody who is going to assist us.'

'Because these days when you need some drugs, you find that they are there. Even though we have shortage of staff you find that you are doing the right thing because you find the right drug there, you manage and then you move to the next patient unlike the previous days.'

[Interviewer]: 'What do you think of the accuracy of the information that we present during these feedbacks?'

[Person 1]: 'Yes, its true, its very true.... so I can say the feedbacks I don't think they try to put us down I think they are doing a good job.... I think we are really improving... even here in the ward....'

[Person 2]: 'Like on the areas we have improved, we feel proud.'

[Person 1]: 'Yes, after listening to the feedbacks, the ones that the team brings to the boardroom, that is better than having these books [written reports] which I will have to read because those books, I don't know, copies are given to the ministry of health.... I think it does not feel good, knowing that these copies are going to the ministry, it feels discouraging a bit.'

### Facilitation within intervention hospitals

Facilitators were either nurses from within the hospital (with a status similar to that of a deputy ward in-charge) or a clinical officer (a non-physician clinician) with training and experience in paediatric care. Their roles and experiences have been described elsewhere [29]. Here we concentrate on what aspects of their role seemed critical to promoting performance improvement amongst the wider set of clinicians and nurses responsible for care in their hospitals. Although not senior personnel within the hospital hierarchy, these actors had a central responsibility for blending the explicit knowledge and expectations encapsulated by the intervention with implicit knowledge of their environment [48]. In this sense, they were formally expected to be the 'earliest adopter' [49], to encourage other early adopters, and to negotiate with or cajole late adopters into delivering correct care. Actions and efforts that characterized successes in this role included: orientation of new staff (establishing a culture) and more widely being available and recognised as a teacher of new skills and use of new tools; agitation for, gaining management approval of, and subsequent implementation of small changes in workflow, procedures, or responsibilities; clear displays of good practice 'at the coal-face' as a role model and advocate for better care; and becoming a visible reminder of the performance expected -- an external conscience.

'Hey, he [the facilitator] is very helpful. You know he is a link between us and the hospital in case there are shortages in terms of supplies; he makes sure we get them or any other problems we are facing. Again he is always there on the forefront sensitizing people when it comes to ETAT even when you see that people are not willing, and then he is also there to arrange for CMEs.'

'In terms of ETAT... and every other time in the HMT [hospital management team] meeting when the managers are there I [the facilitator] am given an opportunity and I give them feedback... I tell them... on this one we are not doing well... on this one we are doing very well... and most of those things are discussed in the meeting and people have given... people chart a way forward on how to overcome some of those things....'

'Like there was a time we came early that morning and there was this child who had been admitted at 5 a.m. with dehydration, had no line because they had tried to get IV access with no success, we gave the child intra-osseous and the child came up. When they were discharged the mother bought us a crate of soda, we felt so nice. The way we worked on that child, [the facilitator] did the intra-osseous, someone else got the fluid, someone fixed an NG tube.....'

'I think that they [facilitators] make sure that work is done... they supervise actually. Like I say if we need oxygen in OPD and it is not there, [the facilitator] will go there and make sure it is availed for any baby who comes, he goes around in nursery supervising and he also coordinates the CMEs and also has lessons on the same.'

'I think if he [the facilitator] were not there, then the... [guidelines] if I can call it that, would not have that meaning, that it has to us. I think we would have just practiced it for a few months and then forgotten all about it.'

'He [the facilitator] does not come as a supervisor, he just comes and works with you all through.'

'During the rounds, I see her [the facilitator] instructing the CO interns on what to do, even on the drugs, the management.'

'...[the facilitator] is... a tank of support and he is... was my conscience when I was working in pediatrics... because may be there were times when I would be tired... maybe I have just finished a ward round and I just want to run away... but then he would remind me.'

So far we have presented thinking on the direct creation of the mediating conditions for practice change linked to the intervention's components and hospitals' management and facilitators. Further downstream effects depended on the ability of local actors to influence internal working routines, commitment, or capabilities (the micro-system), or were influenced by the actual nature of the task required of health workers. These are now discussed.

### Teams, competencies, responsibilities, and expectations

A recurring feature of observations within these hospitals, experiences in other similar hospitals not enrolled in the study, and a theme clearly supported by data was the finding that formal clinical team working was largely absent pre-intervention. Links between cadres (doctors, clinical officers, and nurses), links between departments (outpatient, paediatric ward, newborn unit) were generally hierarchical and perfunctory. Within the intervention sites, training, problem identification and solving, supervision, and facilitation all implicitly emphasized the need for more integrated service provision, although no recipe for achieving this was provided. Where more integrated working evolved, it appeared to be associated with greater satisfaction, a greater sense of team responsibility, greater organizational citizenship behavior, and growing reciprocity and trust. Within intervention hospitals, facilitators were often central to this process, but their achievements seemed dependent on at least the endorsement of senior managers and, for greater success, more active support from management to recognize good performance and help solve resource constraints. Some examples in control hospitals also indicated that engaged managers had the capacity to foster such changes, although successes were more limited. For example, engaged outpatient managers achieved excellent performance for clinical documentation of admissions in one control hospital. However, in the same hospital paediatric drug prescribing remained very poor.

*Commenting on past relationships*

'Well we only meet as cadres.... Like you will find that there is a nurses' meeting, or a clinical officers' meeting but for all those five years I have never seen an OPD (outpatient department) meeting.... I have never.'

'... between the COs and the nurses there is even hate-love relationship over time, the COs and the MOs have the kind of relationship that is pull and push always. So I can't call it a dream team, there is no team, we work together but there is no system of working.'

*Transforming teams, competence, trust, and reciprocity*

'... before the guidelines, you would deal with your emergencies alone, we didn't have an emergency room or emergency drugs in the consultation room, so we used to lose so many patients because of running around trying to resuscitate the patients. We did not have oxygen in the OPD, but now we have it and things are better.'

'Through the efforts of the administration we were able to secure heaters, oxygen concentrators, ambubags, also in nursery, for the admitted pre-terms with sick mothers or no immediate family, we asked the administration whether we can have Nan or lactogen [formula feeds] and they availed it. Sometimes the Med Supt would come to the ward to see whether we are using the PAR forms [job aides] and make sure that things were in line and also for CSF analysis in the lab, people never used to do it, because they used to set it aside and leave it like that but through the pressure of the Med Supt's office they are doing it and you even get the results in 24 hours.'

'Let me give you an example... we supposed to have this glycerin alcohol-based hand wash, in fact there was no glycerin but there was a lot of alcohol in the hospital, so one matron had to buy the glycerin. And we put it in to practice, after every patient that you see you wash, and even the person who are doing rounds with is the matron who bought it, she carries it around so that she makes sure that even if you forget, its not in her presence, so there is a lot of motivation.'

'You see we work as a team, because like if we admit a patient, then that patient will end up in the ward. So when the feedback is given then you will know its the problem in the IMCI [outpatient clinic] or is it in the ward and then that way, you will find a level ground on how the problem will be solved so its good when we work as a team.'

'Usually how the emergencies are handled... like if you get an emergency here, you get that everybody is involved, the nurses, the lab people are there. So we just handle it as a department and we do it perfectly....'

'What I can say is that this place is very busy and I love working with the COs because they are good and they know their work; there are times I stay here [until] 4:30 [p.m.] without going for lunch, so I sacrifice my time because I like the place so much but my colleagues do not like it because they find it too busy.'

[Facilitator]: 'One thing, it has taught me how to network with people, that one is for sure. This programme has made me be a team builder, before, I just used to make sure that everything that I do, I do it right; but when I became a facilitator, it dawned on me that I have to make the other person do it perfectly. So it has made me be a team player to ensure that other people do it right. So I came from being an individual to interacting with the other people to talking to the clinicians, talking to the other nurses, getting very close to the administration especially getting things done'

'Sometimes a patient comes having been prescribed drugs in full and then the BS (malaria test) comes positive and the child is not alert, and there is no quinine (prescribed), and that is at night. The next morning you will come and find that the nurses have already prescribed and even started the treatment.'

Consistent with ideas around change and adoption of new practices [49,50], workplace performance [43], and planned behavior [41], health workers must not only know how to perform a task (*e.g*., prescribing) but be willing to perform it. The intention to act and consequential behavior seemed, from our findings, to be affected by: how cognitively simple the task became, perhaps because of its innate simplicity, or through repetition or training; the degree of effort required to perform the task, linked often to its simplicity; the directness of control over the full execution of the task, linked to trust in colleagues' or team members' co-performance and resource availability; positive experiences of better outcomes; and whether the task was considered a core, personal responsibility or a good fit with existing routines, both linked to the ability of managers or facilitators to change the micro-system if required.

*Task simplicity, effort and outcomes*

'Initially we had problems because you see, when you start new things its really difficult but as time goes by and you have changed your attitudes it becomes simple, you get used to it.'

'Well actually what has kept me going is the results... the changes that are brought from the management of these children in the wards.'

'Because the drugs are not put there especially the anti-malarial Coartem, its really restricted, even in the ward. Initially if a patient had a Coartem prescription, you have to take it to the pharmacy to get it, its not just left to the wards to get access to it. Some will take it and sell it to make money, so the pharmacy really controls it...'

'You see as clinicians we the ones who have a say in terms of clinical care, a nurse cannot prescribe something like multi-vitamins or folate, Vit.A, and we are the ones who calculate the feeds but that was something that was not being done. So that one I realized we had failed in our responsibilities.'

'Feeding used to be just like kienyeji [just anyhow]... actually not knowing ile [the] amount utapeana [you'll give] but now we have a guideline to show... and with the drugs the same... drugs were being prescribed with any dose lakini [but] now there's a standard... there's always a guideline there to show... something else like resuscitation... resuscitation was history... and now we've started resuscitation in nursery and in theatre after delivery... so it means since we started with ETAT+ we have been able to minimize the number of admissions... now this resuscitation is very effective in labor ward and also in theatre... so it means we used to have so many babies in nursery oh... poor score... poor score... poor score... but now they are very few...'

*The extra mile--contrasting attitudes to conducting a lumbar puncture to investigate meningitis*

'I remember even [two named clinicians] are doing LPs in the IMCI [walk-in clinic], every child who comes requiring an LP, it is done and the specimen is sent to the lab directly and they are CO's.... [but]... the CO in charge [says] there is no room, no place where the LPs can be done so I do not understand what he imagines he needs to do an LP, I do not know what course he needs to do an LP.'

'There are people in this hospital that have actually done us proud and if the other people would approach the programme the way they have done, we would be up there. One of them is positive, motivated, ready to work with other people she is not the kind of lady that you will tell, 'I am the in charge here and you cannot do LPs here?' She will tell you, 'I will do an LP because this child needs it and as long as I have all the tools and the right environment, I do not see why not. I was taught in class and I know I am able to do it, ' then she does.'

*Core responsibilities and capability*

[Person 1]: 'The other thing that ETAT has really helped in is in the management of emergencies, because you know before ETAT came, nothing was an emergency to us. We just used to admit, you don't even care, as long as you have made your diagnosis and the patient is taken to the ward. So with ETAT you take a lot of time to find out what exactly is wrong with the child, like last week we had a very sick neonate who had all these shock, hypothermia, hypoglycemia, she was cyanosed all over. So we told the other patients to hold on and we...'

[Person 2]: 'We resuscitated the baby in fact, she was taking the last breath...'

[Person 1]: 'We resuscitated the baby for three hours, even the pediatrician was so happy. So we took the child to the ward and then later on she was referred to Kenyatta [the national hospital]. So you see when I flashback I see that we have lost so many patients in the past, just because of ignorance but with this ETAT at least now we go an extra mile to save a life. By the way at the end of the day I feel happy because I know that I saved a life...'

Two examples of correct prescriptions help illustrate some of these issues. Major changes were achieved in modernizing prescribing practices for a commonly used drug, gentamicin. This required a relatively simple switch in practice by clinicians, supported by job aides and training, when using a drug they might prescribe as a core clinical role to several patients in a single day. The practice change was of potential benefit to nursing colleagues because the frequency of administration was reduced and changing prescribing habits required no alteration to routines or the micro-system. In contrast, prescribing milk-based feeding regimens for severely malnourished children is more complex, although also supported by job aides, and is a task less frequently undertaken. Further, availability of milk feeds and nursing capacity to administer them was often limited, reducing trust in their delivery, and traditionally 'feeding' had often been the preserve not of clinicians but of nurses and, if available, nutritionists. Correct feeding prescription practices were not as easily achieved, or not achieved at all in several hospitals, and even where progress was made, it was often slow and only in response to continued feedback and discussion.

## Discussion

Health systems must be improved if continued and sustained gains in health status are to be made in low-income countries. Research should help guide health system changes. However, such research remains relatively uncommon [51], and specific work on rural African hospitals is unusual. Furthermore, the methods for conducting such work are evolving. Increasingly, it is appreciated that using mixed or multiple methods is desirable to understand complex interventions [19,20,30]. However, the design implications of mixing methods and approaches to integrating findings are challenging [33], with perhaps few groups in low-income settings, including ourselves, claiming real expertise.

So, have we advanced our field of enquiry? Our founding, quantitative study, despite its modest size, is the first to provide credible evidence that a multi-faceted intervention strategy can change provider behaviours and improve the quality of inpatient care across a range of high mortality, target diseases in rural Kenyan settings [24]. Such findings are consistent with recent reports of the effects of multi-faceted interventions in middle- and high-income countries [31,52]. Yet, the intervention we studied produced effects that varied, often substantially, across hospitals and across indicators. Anticipating a need to understand intervention effects, we pre-planned parallel work to characterize hospital contexts [26]. These varied, in part attributable to study selection criteria, and were characterized by high staff turnover [24]. We also found in all settings that health worker motivation was a challenge [27] with, to the degree that it can be measured, perhaps only marginal differences between sites [53]. The components of the intervention were documented and delivered with reasonable consistency [29] but multiple potential barriers to initial uptake of new practices were identified [28]. Other than group assignment, we were unable to explore, statistically, associations of basic, observable hospital characteristics with variation in assessed performance because of our small sample of hospitals [24]. Other approaches are therefore required to help explain marked variation.

To help explain variation in implementation success, we attempted to understand how the 'software' [42] responsible for delivering practice changes (the implementing and hospital personnel) helped produce intervention effects. Our approach, using multiple quantitative and qualitative data sources, bears similarity to one recently recommended in mixed methods research [37], to aspects of realistic evaluation [46,54,55], and to forms of largely deductive cross-case analysis. The deductive approach used may have constrained our ability to identify truly novel mechanisms of effect because initial hypotheses stemmed from our existing knowledge and understanding of theory and Kenyan public hospital settings and active participation in the intervention. Acknowledging this standpoint, we suggest the broad body of data support notions that the nature of the task, the clinical micro-system, the interactions between hospital managers and front-line workers, and the interactions between external implementers and hospital staff are all important in influencing the magnitude of intervention effects. These findings are consistent with multi-level theories of change [17], complexity [56], and the need for a more systems view of intervention design [18]. More specifically, we also examined our intervention and explanations for implementation success against a recently developed, multi-level, consolidated implementation framework [47] (see additional file 1). This framework includes 39 constructs in five major domains. Elements of our multifaceted intervention could be mapped onto all but one of these constructs, and for 23 of them a positive relationship between construct-related activity and implementation success is likely. However, for six constructs we identified no related effect mechanism; for one construct, effects varied with required task, and for nine constructs a relationship between the construct and implementation success was not clear, often because of limitations with the data available to us. Such findings suggest the consolidated framework may be very valuable in guiding future mixed-methods research on implementation in other low-income settings.

Previous work on changing healthcare provider behaviours or performance in low-income settings has spanned training, barriers and attitudes, job aides, incentives, accountability, motivation, and management and supervision amongst others, but often as specific issues [4,42,43,54,57,58]. Our data also highlight the potential cross-cutting influence of a local facilitator as a mechanism to continuously 'update' the software to achieve implementation success. We consider this role--that conceptually has some similarity to that of mid-level managers [59]--particularly worthy of further study. However, it should be remembered that the facilitators were not an isolated intervention, they were embedded in an organization and linked to a wider support supervision framework.

In higher income settings, multi-disciplinary evaluations of complex interventions or mixed methods studies with integrated analyses are receiving increasing attention as a means of producing more nuanced understanding of broader interventions [19,20]. Further promising approaches employ realistic evaluation [46], also used with some success in low-income settings [54,55]. However, as in many areas, we find the technical and financial capacity to undertake such forms of research is often limited in settings such as Kenya. Our limited experience may explain why the synthesis we present here falls somewhere between a trial complemented by qualitative work and a formal mixed or multi-method research study. Yet, if inter-disciplinary health systems research is to expand and be undertaken by researchers from low-income settings themselves, specific efforts will be needed to foster its development.

In conclusion, the body of work we have reported is of a type rarely attempted in low-income settings. The optimum methods for such work may be contested, and we acknowledge likely limitations of those we employed and the potential advantages and disadvantages of our role within the founder study itself. Despite this, we feel confident in stating that the intervention truly worked, but not all the time and not for every practice. Further, we provide a plausible framework drawn from multiple sources of data and lived experience that offers an explanation of how the intervention produced effects.

## Competing interests

The authors have no specific competing interests, but ME and GI were involved in the development of the intervention approach, providing initial training and support supervision. All authors were employed with funds awarded for the conduct and evaluation of the intervention.

## Authors' contributions

ME conceived of the idea for the manuscript and was responsible for the approach to and conduct of analyses before preparing a first draft. All authors provided data and/or reviewed the analytical findings, contributed to the development of the conceptual framework, and provided input to preparation of and approval for the final version of the report.

## Supplementary Material

Additional file 1 **Design of the founder study--a multifaceted intervention trial**. Click here for file

Additional file 2 **Pre-planned parallel studies examining contexts and intervention delivery**. Click here for file

Additional file 3 **Mapping Multifaceted Intervention Approach Characteristics onto the Comprehensive Framework for Advancing Implementation Science**. Click here for file
