# Supplementary material for: Explaining the effects of a multifaceted intervention to improve inpatient care in rural Kenyan hospitals -- interpretation based on retrospective examination of data from participant observation, quantitative and qualitative studies
Source: Implement Sci. 2011 Dec 2;6:124. doi: 10.1186/1748-5908-6-124 (PMC3248845; doi:10.1186/1748-5908-6-124)
Supplement: Additional file 1 — Design of the founder study--a multifaceted intervention trial. [file 1748-5908-6-124-S1.DOCX]

**Additional File 1: Design of the founder study—a multifaceted intervention trial**

|  | The hospital intervention trial was conducted in eight district hospitals, initially identified on the basis of five district-specific criteria and hospital workload statistics requiring a minimum of 1,000 paediatric admissions and 1,200 deliveries per year [21, 26]. In brief, the parent study was a parallel group, controlled intervention study with hospitals randomized into full (n = 4) or partial (referred to as control, n = 4) intervention groups [26]. The parent study design, hospital locations, the intervention and the study contexts and a full description of trial results are described in detail elsewhere [26].  The full intervention components delivered to hospital H1 to H4 included: setting up a scheme for regular hospital assessment through surveys conducted six monthly, followed by face to face feedback of findings in intervention sites, and written feedback for both hospital groups; 5.5 day training aimed at 32 health workers of all cadres approximately six to ten weeks after baseline surveys (July to August 2006) in intervention hospitals; provision of clinical practice guidelines and job aides introduced with training; an external supervisory process; and identification of a full-time local facilitator (a nurse or diploma-level clinician) responsible for promoting guideline use and on-site problem solving. Supervision visits were approximately two to three monthly, but facilitation remained in place throughout the 18 months. The package for partial intervention or control sites (H5 to H8) included five components: six-month surveys with written feedback only; provision of clinical practice guidelines and job aides; and a 1.5-day initial guideline seminar for approximately 40 hospital staff. The design thus compared two alternative intensities of intervention although we refer to one arm as the ‘control.’ Intervention effectiveness was assessed by comparing performance of hospitals in the two groups at 18 months **after** initiation of intervention; this was done both in a cross-sectional analysis and by examining changes from a pre-intervention baseline by 18 months [24].  ***Performance Indicators***  Primary effectiveness measures were 14 process indicators measured on pediatric admissions aged 2 to 59 months at 18 months post baseline (survey four). Secondary measures were four valued system outcomes of admission and changes in structure measured at the hospital level.The trial was not designed to evaluate mortality effects. |
| --- | --- |
