# Supplementary material for: Explaining the effects of a multifaceted intervention to improve inpatient care in rural Kenyan hospitals -- interpretation based on retrospective examination of data from participant observation, quantitative and qualitative studies
Source: Implement Sci. 2011 Dec 2;6:124. doi: 10.1186/1748-5908-6-124 (PMC3248845; doi:10.1186/1748-5908-6-124)
Supplement: Additional file 2 — Pre-planned parallel studies examining contexts and intervention delivery. [file 1748-5908-6-124-S2.DOCX]

**Additional File 2**. **Pre-planned parallel studies examining contexts and intervention delivery**

|  | Training and guidelines may only result in changes in the provision of care in settings with adequate physical and human resources. Supervision and feedback may have little effect if staff and management are preoccupied with other priorities, while a specific intervention effect might be hidden by any broad, major health sector developments. At a local level, the intervention delivered may work to different degrees in hospitals of different sizes or those that lose key personnel or trained staff. Studies aimed, therefore, to describe the hospitals and the health system as contexts within which this multi-faceted intervention was delivered, while exploring health worker motivation as a potentially important modifying factor and specific barriers to uptake of new practices. Specific studies included:  ***Study one:*** Intended to describe relevant health policy and institutional environment and changes over the 18-month intervention and study hospitals in terms of human and material resource capacity and indicate nature (quality) of care provided for children and newborns at baseline [26].  ***Study two:*** Intended to explore health worker motivation in study hospitals prior to any intervention as motivation is considered to be a potentially important modifier of implementation success [27].  ***Study three:*** Intended to describe, from the health workers’ perspective, factors that may prevent broad use of the guidelines with data collection undertaken four to five months after initiating the intervention [28].  ***Study four:*** Intended to describe how the intervention was actually delivered over the 18 months period and explore health workers views of different intervention aspects after 16-18 months of intervention experience [29]. |
| --- | --- |
