# Supplementary material for: Explaining the effects of a multifaceted intervention to improve inpatient care in rural Kenyan hospitals -- interpretation based on retrospective examination of data from participant observation, quantitative and qualitative studies
Source: Implement Sci. 2011 Dec 2;6:124. doi: 10.1186/1748-5908-6-124 (PMC3248845; doi:10.1186/1748-5908-6-124)
Supplement: Additional file 3 — Mapping Multifaceted Intervention Approach Characteristics onto the Comprehensive Framework for Advancing Implementation Science. [file 1748-5908-6-124-S3.DOCX]

**Appendix 1.** Mapping the full package of the multifacteted intervention against the Consolidated Framework for Implementation Research [47] and indication of whether activity within a construct promoted implementation success (1), had little perceived effect on implementation success (0) or whether the situation was unclear.

| **Consolidated Framework for Implementation Research Domains and Constructs** | **Full Intervention Package Characteristics** | **Perceived Effect on Implementation Success** |
| --- | --- | --- |
| **I. INTERVENTION CHARACTERISTICS** |  |  |
| **A Intervention Source** Perception of key stakeholders about whether the intervention is externally or internally developed. | External | 1 |
| **B Evidence Strength & Quality** Stakeholders’ perceptions of the quality and validity of evidence supporting the belief that the intervention will have desired outcomes. | Credible and authoritative | 0 |
| **C Relative advantage** Stakeholders’ perception of the advantage of implementing the intervention versus an alternative solution | Not clear | Not clear |
| **D Adaptability** The degree to which an intervention can be adapted, tailored, refined, or reinvented to meet local needs | Guidelines not adaptable but initial development tailored and mode of implementation adaptable | 1 |
| **E Trialability** The ability to test the intervention on a small scale in the organization [8], and to be able to reverse course (undo implementation) if warranted. | No | 0 |
| **F Complexity** Perceived difficulty of implementation, reflected by duration, scope, radicalness, disruptiveness, centrality, and intricacy and number of steps required to implement | Varied for different guidelines | 0 - 1 |
| **G Design Quality and Packaging** Perceived excellence in how the intervention is bundled, presented, and assembled | Meaningful, guideline format still a challenge | 1 |
| **H Cost** Costs of the intervention and costs associated with implementing that intervention including investment, supply, and opportunity costs. | Not clear what costs of change were to hospitals, were opportunity costs in the form of time to learn new practices | Not clear |
| **II. OUTER SETTING** |  |  |
| **A Patient Needs & Resources** The extent to which patient needs, as well as barriers and facilitators to meet those needs are accurately known and prioritized by the organization. | Not clear |  |
| **B Cosmopolitanism** The degree to which an organization is networked with other external organizations. | Promoted through shared feedback | 1 |
| **C Peer Pressure** Mimetic or competitive pressure to implement an intervention; typically because most or other key peer or competing organizations have already implemented or in a bid for a competitive edge. | Chance to be leading centres and some shared feedback of performance in other hospitals | 1 |
| **D External Policy & Incentives** A broad construct that includes external strategies to spread interventions including policy and regulations (governmental or other central entity), external mandates, recommendations and guidelines, pay-for-performance, collaboratives, and public or benchmark reporting. | Endorsed by government but no incentives | Not clear |
| **III. INNER SETTING** |  |  |
| **A Structural Characteristics** The social architecture, age, maturity, and size of an organization. | Hospitals shared hierarchical structure but nature of organisation varied | Not clear |
| **B Networks & Communications** The nature and quality of webs of social networks and the nature and quality of formal and informal communications within an organization. | Limited professional networks, poor internal communications that intervention attempted to address | 1 |
| **C Culture** Norms, values, and basic assumptions of a given organization. | Developing a culture of better practice an aim of intervention | 1 |
| **D Implementation Climate** The absorptive capacity for change, shared receptivity of involved individuals to an intervention and the extent to which use of that intervention will be rewarded, supported, and expected within their organisation | Intervention promoted personal and shared responsibility | 1 |
| **D**.**1 Tension for Change** The degree to which stakeholders perceive the current situation as intolerable or needing change. | Intervention aimed to demonstrate that better practice possible despite resource challenges | 1 |
| **D.2 Compatibility** The degree of tangible fit between meaning and values attached to the intervention by involved individuals, how those align with individuals’ own norms, values, and perceived risks and needs, and how the intervention fits with existing workflows and systems. | Providing 'modern' care and being able to do this within existing routines part of intervention's aim | 1 |
| **D.3 Relative Priority** Individuals’ shared perception of the importance of the implementation within the organization. | Need to improve paediatric care appreciated after baseline survey and training | 1 |
| **D.**4 **Organizational Incentives & Rewards** Extrinsic incentives such as goal-sharing awards, performance reviews, promotions, and raises in salary and less tangible incentives such as increased stature or respect. | Intervention aimed to promote positive sense of achievement for successes, no specific incentives | 1 |
| **D.5 Goals and Feedbac**k The degree to which goals are clearly communicated, acted upon, and fed back to staff and alignment of that feedback with goals. | Feedback on performance part of intervention | 1 |
| D.**6 Learning Climate** A climate in which: a) leaders express their own fallibility and need for team members’ assistance, and input; b) team members feel that they are essential, valued, and knowledgeable partners in the change process; c) individuals feel psychologically safe to try new methods; and d) there is sufficient time and space for reflective thinking and evaluation. | Aim to promote team-working but constraints on time / space / support although facilitator supported change efforts | Not clear |
| **E Readiness for Implementation** Tangible and immediate indicators of organizational commitment to its decision to implement an intervention | Indicators of commitment only developed after initiation of intervention | 0 |
| **E.1 Leadership** Engagement Commitment, involvement, and accountability of leaders and managers with the implementation. | Considerable variability between hospitals | Not clear |
| **E.2 Available Resources** The level of resources dedicated for implementation and on-going operations including money, training, education, physical space, and time. | Very limited resources to support implementation at hospitals | 0 |
| E.**3 Access to knowledge and information** Ease of access to digestible information and knowledge about the intervention and how to incorporate it into work tasks. | Other than guidelines and access to facilitator limited access to wider information | 0 |
| **IV. CHARACTERISTICS OF INDIVIDUALS** |  |  |
| **A Knowledge & Beliefs about the Intervention**, Individuals’ attitudes toward and value placed on the intervention as well as familiarity with facts, truths, and principles related to the intervention. | Very mixed initial responses to guidelines and intervention | Not clear |
| **B Self-efficacy** Individual belief in their own capabilities to execute courses of action to achieve implementation goals | Self-efficacy observed to emerge with familiarity and support from facilitator | 1 |
| **C Individual Stage of Change** Characterization of the phase an individual is in, as he or she progresses toward skilled, enthusiastic, and sustained use of the intervention. | Very large number of clinicians so overall assessment difficult | Not clear |
| **D Individual Identification with Organisation** A broad construct related to how individuals perceive the organization and their relationship and degree of commitment with that organization. | Low commitment to organisation at the start, intervention aimed to promote commitment to better care through participation at all levels of organisation | 1 |
| **E Other Personal Attributes** A broad construct to include other personal traits such as tolerance of ambiguity, intellectual ability, motivation, values, competence, capacity, and learning style. | Guidelines designed to fit competencies and be clear | 1 |
| **V. PROCESS** |  |  |
| **A Planning** The degree to which a scheme or method of behavior and tasks for implementing an intervention are developed in advance and the quality of those schemes or methods. | Intervention aspects of supervision, feedback and facilitation were newly developed | 0 |
| **B Engaging** Attracting and involving appropriate individuals in the implementation and use of the intervention through a combined strategy of social marketing, education, role modeling, training, and other similar activities. | Use of training of a 'critical mass' of staff involved in paediatric care and regular meetings with administrators and staff | 1 |
| **B.1 Opinion Leaders** Individuals in an organization who have formal or informal influence on the attitudes and beliefs of their colleagues with respect to implementing the intervention | Role of facilitator and some senior staff as opinion leaders | 1 |
| **B.2 Formally appointed internal implementation leaders** Individuals from within the organization who have been formally appointed with responsibility for implementing an intervention as coordinator, project manager, team leader, or other similar role. | Facilitator | 1 |
| **B.3 Champions** “Individuals who dedicate themselves to supporting, marketing, and ‘driving through’ an [implementation]” [101](p. 182), overcoming indifference or resistance that the intervention may provoke in an organization. | Facilitator often able to draw on an informal group of supporters including clinicians and nurses | 1 |
| **B.4 External Change Agents** Individuals who are affiliated with an outside entity who formally influence or facilitate intervention decisions in a desirable direction. | Supervisors from research institution and University | 1 |
| **C Executing** Carrying out or accomplishing the implementation according to plan. | Structured approach to supervision and feedback over extended period | Not clear |
| **D Reflecting & Evaluating** Quantitative and qualitative feedback about the progress and quality of implementation accompanied with regular personal and team debriefing about progress and experience. | Implementation (research team) had frequent but informal meetings to discuss progress and different approaches used across settings | Not clear |
